# Compliant Titin Isoform Content Is Reduced in Left Ventricles of Sedentary Versus Active Rats

**DOI:** 10.3389/fphys.2020.00015

**Published:** 2020-02-05

**Authors:** Charles S. Chung, Mark A. Hiske, Arjun Chadha, Patrick J. Mueller

**Affiliations:** Department of Physiology, Wayne State University, Detroit, MI, United States

**Keywords:** heart, diastole, passive stiffness, exercise, inactivity, titin

## Abstract

A sedentary lifestyle is associated with increased cardiovascular risk factors and reduced cardiac compliance when compared to a lifestyle that includes exercise training. Exercise training increases cardiac compliance in humans, but the mechanisms underlying this improvement are unknown. A major determinant of cardiac compliance is the compliance of the giant elastic protein titin. Experimentally reducing titin compliance in animal models reduces exercise tolerance, but it is not known whether sedentary versus chronic exercise conditions cause differences in titin isoform content. We hypothesized that sedentary conditions would be associated with a reduction in the content of the longer, more compliant N2BA isoform relative to the stiffer N2B isoform (yielding a reduced N2BA:N2B ratio) compared to age-matched exercising controls. We obtained left ventricles from 16-week old rats housed for 12 weeks in standard (sedentary) or voluntary running wheel (exercised) housing. The N2BA:N2B ratio was decreased in the hearts of sedentary versus active rats (*p* = 0.041). Gene expression of a *titin* mRNA splicing factor, RNA Binding Motif 20 protein (RBM20), correlated negatively with N2BA:N2B ratios (*p* = 0.006, *r* = −0.449), but was not different between groups, suggesting that RBM20 may be regulated post-transcriptionally. Total phosphorylation of cardiac titin was not different between the active and sedentary groups. This study is the first to demonstrate that sedentary rats exhibit reduced cardiac titin N2BA:N2B isoform ratios, which implies reduced cardiac compliance. These data suggest that a lack of exercise (running wheel) reduces cardiac compliance and that exercise itself increases cardiac compliance.

## Introduction

Sedentary behavior is a major risk factor for premature death, and prolonged periods of sedentary behavior are considered a medical hazard (Blair et al., 1995; Bassett et al., 2010; Lavie et al., 2019). Sedentary behavior is also associated with reduced cardiac compliance (inverse of passive stiffness), which itself is an important index of cardiac health (Lalande et al., 2017; Howden et al., 2018). Cardiac compliance is primarily determined by titin, the giant elastic protein present in the sarcomeres of striated muscles, including cardiac myocytes (Granzier and Irving, 1995; Granzier and Labeit, 2004; Chung and Granzier, 2011). Increasing titin’s compliance reduces the slope of the pressure–volume relationship during diastole, providing the heart with a greater capacity to fill during increases in venous return that occur during exercise (Lalande et al., 2017). Reducing titin compliance (increasing passive stiffness) impairs diastolic filling and the ability of the left ventricle to increase stroke volume during exercise.

Titin is encoded by a single gene (*titin*, *ttn*), but can be alternatively spliced. Increased splicing of *titin* mRNA reduces the length of the elastic region of the protein, which resides in the I-band of the sarcomere (Granzier and Labeit, 2004; Guo et al., 2012). Alternative splicing of *titin* is an important mechanism to modify titin’s properties and thus cardiac compliance (Granzier and Labeit, 2004). Cardiac muscle expresses two classes of splice isoforms: the longer, more compliant N2BA isoform and the shorter, less compliant N2B isoform. The two isoforms are distributed throughout the myofibrils so that the ratio of their relative contents defines the compliance of a given myocyte (Trombitas et al., 2001). Increases in the relative content ratio of the N2BA to N2B isoform (N2BA:N2B) is associated with increased ventricular compliance. The relative content of these two isoforms is primarily controlled by RNA Binding Motif 20 protein (RBM20) (Guo et al., 2012, 2013). An increased RBM20 content is consistent with a reduced N2BA:N2B ratio, as additional exons of *titin* mRNA are spliced out to form the shorter, stiffer, N2B protein isoform (Guo et al., 2012, 2013; Methawasin et al., 2016; Zhu et al., 2017). RBM20’s activity may be inhibited by the Polypyrimidine Tract Binding protein 4 (PTB4) (Dauksaite and Gotthardt, 2018). Thus, multiple mechanisms to modify titin isoform content have been proposed.

While our current knowledge of the relationship between titin and exercise tolerance is limited, experimentally reduced titin compliance is associated with reduced exercise tolerance (Lalande et al., 2017). This relationship is based primarily on studies using transgenic and knockout mouse models that modified the size of the *titin* gene to modify titin compliance. Reducing RBM20 expression or activity has been shown to increase the content of compliant titin isoforms and increase exercise tolerance (Methawasin et al., 2016; Slater et al., 2017). In humans, there are fewer studies directly relating N2BA:N2B ratios and exercise tolerance. In heart failure patients with a reduced ejection fraction, exercise tolerance correlated positively (*r* = 0.8) with the N2BA:N2B ratio but not with ejection fraction (Nagueh et al., 2004). This correlation was possible only because the myocardial tissues were made available for biochemical study after an explant of a heart during transplant. The remainder of the human studies use measures of cardiac compliance without direct measure of titin properties but report a stronger correlation between exercise tolerance and cardiac compliance than between exercise tolerance and heart failure status (Meyer et al., 2004). While these data imply that titin compliance predicts exercise tolerance, there are no data to indicate whether sedentary versus physical activity conditions result in differences in titin isoform content.

In humans, exercise training increases both exercise tolerance (Edelmann et al., 2011; Fujimoto et al., 2012) and clinical measures of cardiac compliance (Howden et al., 2018). These data indicate that cardiac compliance differs between sedentary and active groups. However, it is unknown if titin isoform content shifted to an increase in the compliant isoform (N2BA) in response to exercise. In mice, moderate duration exercise protocols (i.e., ≤1 month) have been reported to modify titin compliance via post-translational modifications but did not appear to alter titin isoform content (Hidalgo et al., 2014; Muller et al., 2014; Slater et al., 2017). It is possible that the short-duration of the studies and the use of mouse models might have masked changes in the N2BA:N2B ratio. Thus, it remains unknown whether sedentary conditions reduce titin compliance (or whether more chronic activity (exercise) increases titin compliance), which would provide a mechanism for exercise training-induced increases in exercise tolerance in sedentary individuals.

This study sought to evaluate titin isoform content in left ventricular tissue from established rat models that promote inactivity or voluntary exercise. The models utilize chronic (12 weeks) voluntary running wheel access to mimic exercise activity and sedentary housing conditions to mimic inactivity (Mischel and Mueller, 2011). In small animal models, standard laboratory conditions are increasingly associated with un-natural sedentary conditions. Sedentary lifestyles reflect a form of inactivity and are considered by some to be a pathophysiologic state associated with numerous diseases including cardiac dysfunction (Booth et al., 2017). Further, voluntary running wheel activity in wild mice is comparable to that of lab-mice (Meijer and Robbers, 2014). These data suggest that sedentary housing conditions for laboratory rodents is actually an unnatural condition, and voluntary running wheel activity would reflect a more natural, control state for the rats. We hypothesized that the titin N2BA:N2B ratio would be lower in rat hearts after 12 weeks of sedentary conditions compared to chronic voluntary wheel running conditions. We also hypothesized that changes in the titin isoform ratio would be dependent on *titin* mRNA splicing factors such as RBM20.

## Materials and Methods

### Animal Model and Samples

Vertebrate animal procedures were approved by the Institutional Animal Care and Use Committee of Wayne State University and conducted in accordance with the American Physiological Society’s “Guiding Principles in the Care and Use of Animals.” Rat samples were collected using an established exercise protocol as previously described (Mischel and Mueller, 2011). Briefly, 75–100 g male Sprague Dawley rats, approximately 4 weeks of age, were obtained (Envigo, Indianapolis, IN, United States). Thirty-nine rats were randomly distributed into two groups. Active rats performing voluntary exercise (EX, *n* = 20) were housed in 9″ × 18″ × 9″ cages containing a commercially available running wheel (Techniplast, Eaton, PA, United States; wheel diameter 47 cm). Bicycle computers (Sigma Sport, Olney, IL, United States) recorded daily and cumulative running distances. Voluntary exercised rats had 24-h access to the running wheel. Rats in the sedentary group (SED, *n* = 19) were housed in identically sized cages that lacked a running wheel, which mimics typical laboratory conditions for rats. All animals were individually housed and received food and water *ad libitum*. After 12 weeks of housing under sedentary or exercising conditions (16 weeks of age), running wheels were removed from the cages of exercising animals 24 h prior to sacrifice to prevent acute exercise from impacting the results (Muller et al., 2014). The rats were sacrificed via decapitation under deep anesthesia (Fatal Plus, 50 mg/kg body weight (BW), i.p.; Vortech, Dearborn, MI, United States). The left ventricle was dissected, weighed, and flash-frozen in a container of isopentane chilled with dry ice and subsequently stored at −80°C. Two segments of left ventricular free wall were isolated under liquid nitrogen for protein analysis (∼15 mg) and RNA isolation (∼150 mg).

### Tissue Solubilization

The small (∼15 mg) segments of the left ventricular wall used for protein analysis were pulverized using Kontes Dounce homogenizers (Kimble Chase, Rockwood, TN, United States) cooled in liquid nitrogen, as previously described (Lahmers et al., 2004; Chung et al., 2013). Subsequently, all samples were solubilized in a solution containing equal amounts of solubilization buffer (8 M Urea, 2 M thiourea, 0.05 M Tris–HCl, 0.075 M DTT, 3% sodium dodecyl sulfate (SDS), and 0.03% Bromophenol Blue all adjusted to pH 6.8) and glycerol with protease inhibitors [50% glycerol, 0.008% Leupeptin, 0.04 mM E-64, and 0.5 mM phenylmethylsulfonyl fluoride (PMSF)] at 60°C. Solubilized samples were centrifuged, aliquoted, flash-frozen in liquid nitrogen, and stored at −80°C.

### Titin Isoform and Phosphorylation Analysis

To determine titin isoform content, 1% Agarose gels (agarose with 0.005 M tris base, 0.039 M glycine, 2% SDS, 30% glycerol) were prepared in a large-format gel system (Hoeffer SE600X, Hoefer, Inc., Holliston, MA, United States) (Lahmers et al., 2004; Chung et al., 2013; Zhu and Guo, 2017). Briefly, solubilized samples were loaded, and gel electrophoresis was run at 15 mA/gel for 3 h and 20 min. Gels were stained using Coomassie Brilliant Blue and scanned using a commercially available scanner (Epson V850, Epson America, Inc., Long Beach, CA, United States) with a calibrated Optical Density step tablet (Stouffer Industries, Mishawaka, IN, United States). The scan was normalized for Optical Density using a custom MATLAB script (MathWorks, Natick, MA, United States) and analyzed using ImageQuant TL (GE Healthcare Bio-Sciences Corp., Marlborough, MA, United States). Relative titin mobility was evaluated by loading and co-electrophoresing rat ventricular and soleus muscle reference samples. Relative mobility was calculated using ImageQuant TL as previously described using rat soleus N2A titin isoform, Nebulin, and Myosin Heavy Chain as mobility references (Granzier and Irving, 1995; Buck et al., 2010; Li et al., 2012). Relative titin isoform content (N2BA:N2B ratio) was evaluated by loading individual samples in a range of five volumes (3–9 μL) at dilutions pre-determined to be within the linear range of detection. Background subtraction was performed using the Rolling Ball function (Sternberg, 1983). Relative content was determined using the slope of the optical density versus volume loading for MHC and all titin bands (N2BA, N2B, and T2, the titin degradation band). Two minor bands representing the N2BA isoform were summed to determine the N2BA content (Freiburg et al., 2000).

To determine the total titin phosphorylation status, 2–12% gradient acrylamide gels (2–12% Acrylamide, (1:50) Bis-acrylamide, 0.1% SDS, 5% glycerol, 10× Fairbanks buffer (400 mM Tris Base, 200 mM Sodium Acetate, 20 mM EDTA, pH adjusted to 7.5 with glacial acetic acid), 0.0625% Ammonium Persulfate, and 0.0875% TEMED) were prepared in Mini-PROTEAN Cassettes (BioRad) and allowed to polymerize. Electrophoresis was pre-run with running buffer (10× Fairbanks buffer, 0.1% SDS, and 11.44 mM βME) at 60 V for 3 min without sample. Solubilized samples were then loaded and gel electrophoresis was run at 60 V for 68 min and then 90 V for 70 min. Gels were fixed overnight in 50% methanol and 10% acetic acid, washed three times using ultrapure water, and stained for total phosphate content using a rapid protocol for Pro-Q Diamond staining (Thermo Fisher Scientific, Waltham, MA, United States). Gels were destained using Pro-Q Diamond Destain Solution and washed twice with ultrapure water before scanning with a Typhoon Trio + scanner at 532-nm excitation and 560-nm emission. Gels were then stained for total protein content with Sypro Ruby Gel Stain using the rapid protocol, washed, and scanned with a Typhoon Trio + scanner at 488-nm excitation and 610-nm emission. Bands were quantified using ImageQuant TL (GE Healthcare Bio-Sciences Corp., Marlborough, MA, United States).

### Rat Cardiac mRNA Expression Analysis

To determine the mRNA expression of titin-splicing proteins RBM20 and PTB, frozen left ventricular tissue segments were placed in pre-chilled (−80°C) RNAlater-ICE (Ambion) and allowed to transition from −80°C to −20°C for at least 16 h. RNA was extracted from samples weighing approximately 150 mg using the Trizol method (Rio et al., 2010). The RNA concentration was measured in duplicate using a Tecan microplate reader and averaged; all samples had a concentration >100 ng/μL or were re-isolated. RT-PCR was performed using an Applied Biosystems 7300 Real-Time PCR System (Foster City, CA, United States). The following custom primers were obtained from Integrated DNA Technologies, Inc (Coralville, IA, United States): *rbm20* Forward: 5′-CTCAGCTCACCCTCCACC-3′; *rbm20* Reverse: 5′-GTTGAAGAGAGGCTGGGAC-3′; *ptb* Forward 5′-AAAGCCTCTTTATTCTCTTCGGCGTCTAC-3′; *ptb* Reverse 5′-TGAAGCCTTTGACCACACCACCGTTGCTGG-3′; β*-actin* Forward: 5′-AGTGCTGTGGGTGTAGGTA-3′; β*-actin* Reverse: 5′-TTTAGATGGAGAAGGGATGAGAC-3′. All samples were run in triplicate for each primer, then averaged. The 2^–ΔΔCt^ method was used to quantify mRNA expression; ΔCt was calculated between *rbm20* or *ptb* and β*-actin* (Livak and Schmittgen, 2001). Fold change was calculated against the SED group. Two samples were excluded from analysis of each primer set because data suggested poor amplification (e.g., average Ct > 20, or 2^–ΔΔCt^ was 3 standard deviations outside the mean of all samples).

### Statistics

Data are presented as individual data points or mean ± SD. Statistics were calculated in SPSS (Ver 25, IBM Corporation, Armonk, NY, United States). Normality was tested using the Shapiro–Wilk test. For normally distributed data sets, unpaired *t*-tests were performed. For non-normally distributed data sets, a Mann–Whitney *U* test was used. Linear regression was also performed. A *p*-value of less than 0.05 was considered significant.

## Results

### Rat Chronic Exercise and Titin

Voluntary exercise rats (EX) ran a mean cumulative distance of 130 ± 47 km over 12 weeks (*n* = 19, excluding one sample that was a statistical outlier with a distance of 607 km over 12 weeks). Table 1 shows body and organ weight data from sedentary and active rats. As expected, the BW differed between groups (*p* < 0.001). The BW was larger in the 16-week old sedentary (SED) versus the exercise (EX) group. Left ventricular weights (LVW) of the SED animals were not statistically different than the EX group (*p* = 0.795). The LVW:BW ratio was significantly larger in the EX group compared to the SED groups (*p* = 0.012).

**TABLE 1:** Physiologic measures from rats.

**Parameter**	**Sedentary control (SED)**	**Exercise (EX)**	***p*-value**
n	19	20	
BW (g)	419 ± 23	379 ± 40	*0.001*
LV Weight (mg)	869 ± 53	828 ± 83	0.079
LV:BW (mg/g)	2.07 ± 0.10	2.19 ± 0.14	*0.012*

*Sample numbers (n) are biological replicates for individual rats. Listed *p*-values for SED versus EX via *t*-test for BW and LVW, Mann–Whitney *U* test for LVW:BW; italics denotes *p* < 0.05. Body weight (BW); left ventricular (LV) weight; LV weight to body weight ratio (LV:BW).*

**FIGURE 1:**
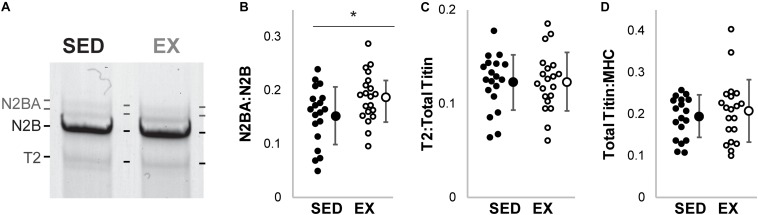
Titin protein analysis. **(A)** Example gel electrophoresis for an exercise and sedentary sample. (Linear contrast adjustment applied to the original image for visualization.) **(B)** The ratio of the longer more compliant N2BA isoform of titin to its shorter N2B isoform (N2BA:N2B) was decreased in hearts from rats housed in sedentary conditions (SED; black circles filled, *n* = 19), versus exercise conditions (EX, open circles, *n* = 20, * denotes *p* = 0.041 by *t*-test). **(C)** Titin degradation (T2) versus total titin (TTN) and **(D)** TTN to Myosin Heavy Chain (MHC) ratio were not different (*p* = 0.95 and *p* = 0.54, respectively) between the SED and EX groups.

Cardiac titin isoform analysis is shown in Figure 1. The N2BA:N2B ratio for the SED group was significantly smaller than the EX group (*p* = 0.041). Relative mobility, which indicates titin size, was not different for either the N2BA nor N2B isoforms in the 16-week groups (*p* = 0.18, data not shown), suggesting otherwise normal titin splicing. The ratio between relative content of degraded titin to total titin (T2:TTN) and the ratio between total titin and myosin heavy chain content (TTN:MHC) were not different between groups.

**FIGURE 2:**
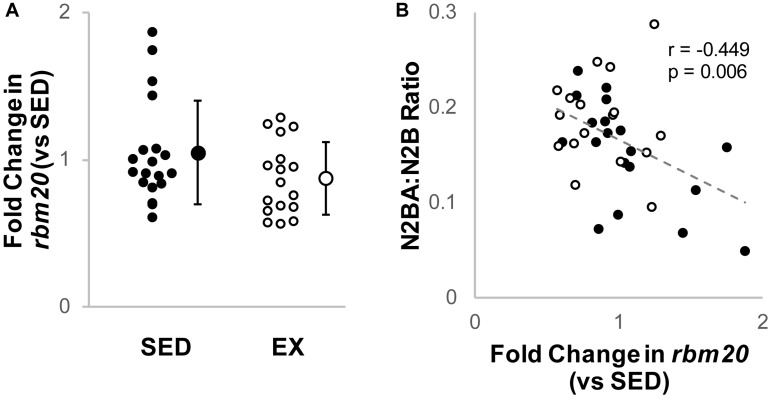
Analysis of Titin Splicing factor RBM20. **(A)** Fold change (2^–ΔΔCt^) in relative *RNA Binding Motif 2*0 (*RBM20*) mRNA expression in the exercise group (EX, open circles, *n* = 17) compared to sedentary controls (SED, black circles, *n* = 19); the difference was not statistically significant (*p* = 0.65 by Mann–Whitney *U* test). Individual samples shown as circles, mean ± SD shown at the right of individual data. **(B)** Titin isoform ratio negatively correlated (gray dashed line, *r* = –0.449, *p* = 0.006, linear regression) with *RBM20* mRNA expression for all samples, which is consistent with prior work that *RBM20* expression is the primary driver of changes in N2BA:N2B ratio.

**FIGURE 3:**
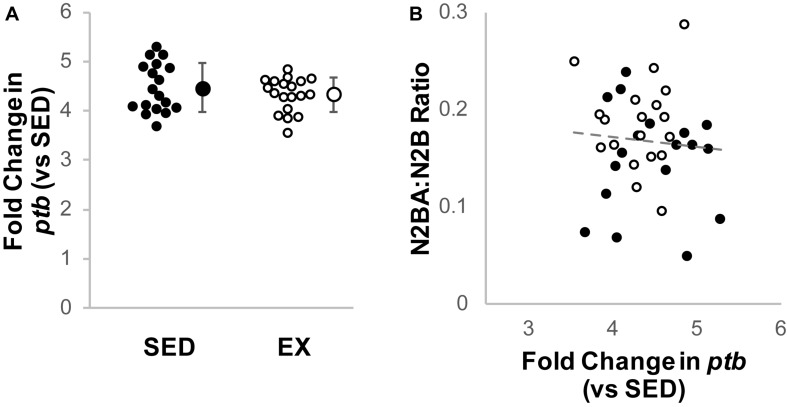
Analysis of titin splicing factor PTB. **(A)** Fold change (2^–ΔΔCt^) in relative *Polypyrimidine Tract Binding protein* (*PTB*) mRNA expression in the exercise group (EX, open circles, *n* = 19) compared to sedentary controls (SED, black circles, *n* = 18); the difference was not statistically significant (*p* = 0.314 by *t*-test). Individual samples shown as circles, mean ± SD shown at the right of individual data. **(B)** Titin isoform ratio did not correlate with *PTB* expression (gray dashed line, *r* = 0.078, *p* = 0.645, linear regression).

Cardiac *rbm20* mRNA expression tended to be less in ventricular tissue from the EX versus SED group (Figure 2A), but this difference did not reach statistical significance (*p* = 0.65 by Mann–Whitney *U* test). However, *rbm20* expression was significantly and negatively correlated with N2BA:N2B ratios for all 16-week samples (*p* = 0.006, *r* = −0.449) (Figure 2B). Cardiac *ptb* mRNA expression was not different between SED and EX groups (*p* = 0.314 by *t*-test) (Figure 3A), and there was no correlation between *ptb* mRNA expression and N2BA:N2B ratios (*p* = 0.645, *r* = −0.078) in the 16 week rat hearts (Figure 3B).

**FIGURE 4:**
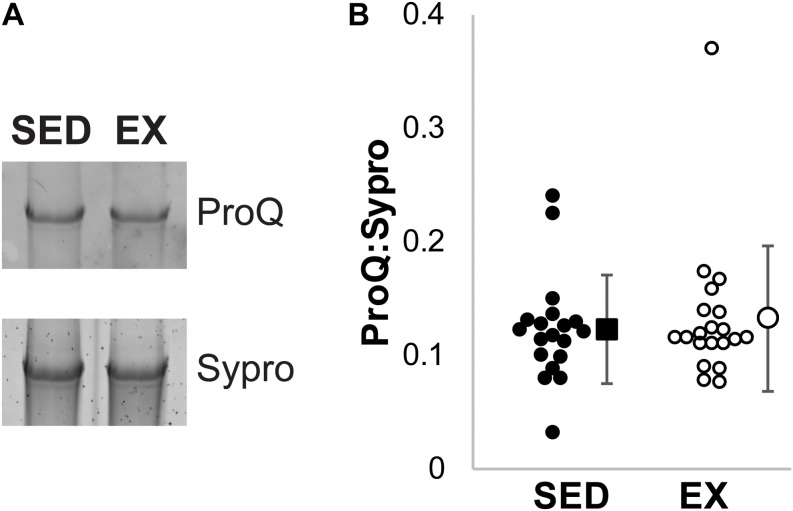
ProQ Diamond Phosphorylation analysis. **(A)** Example ProQ Diamond and Sypro Ruby staining of titin, contrast adjusted. **(B)** The total phosphorylation to protein ratio was not statistically significant (*p* = 0.95 by Mann–Whitney *U* test) between the SED (black filled, *n* = 19) and EX (open circles, *n* = 20) groups. Individual samples shown as circles, mean ± SD shown at the right of individual data.

Total phosphorylation of titin was evaluated using ProQ Diamond staining (Figure 4). No significant difference was observed in the total phosphorylation of titin between the exercised and sedentary groups (*p* = 0.95 by Mann–Whitney *U* test).

## Discussion

Sedentary aging is associated with an increase in both cardiovascular and general health risks (Blair et al., 1995; Bassett et al., 2010; Booth et al., 2017; Lavie et al., 2019) and reduced cardiac compliance (Howden et al., 2018). Exercise is thought to alleviate these changes and increase cardiac compliance (Howden et al., 2018). To date, the mechanisms underlying exercise-dependent increases in cardiac compliance have not been fully elucidated. The current study is the first to show that the titin N2BA:N2B ratio, which correlates with cardiac compliance, is reduced in a sedentary animal model.

### Titin Isoform Content

Titin is well known to be the major determining factor of cardiac passive stiffness (Granzier and Irving, 1995; Chung and Granzier, 2011) and is known to be able to predict exercise tolerance (Lalande et al., 2017). It is less well understood how exercise impacts titin’s compliance. Specifically, there have been only limited studies on how short-term (≤4 weeks) differences in activity/exercise modifies titin, despite evidence that exercise increases myocardial compliance (Edelmann et al., 2011; Howden et al., 2018).

In animal models, the relationship between titin and exercise has historically been focused on how titin compliance can predict exercise tolerance (Lalande et al., 2017). For example, when transgenic methods were used to delete exons that code for several proximal Ig domains of the I-band of titin, titin’s passive stiffness increased by approximately 25% (Chung et al., 2013). This reduced compliance was coincident with a 30% reduction in exercise tolerance. More recent studies have further confirmed that reduced titin compliance (increased stiffness) leads to reduced exercise tolerance; whereas, increased titin compliance increases exercise tolerance (see Lalande et al., 2017 for a recent review). Titin isoform expression (and thereby compliance) has also been modified by interfering with the *titin* mRNA splicing factor RBM20. A reduction in RBM20 content or RBM20 activity increases the content of the larger N2BA isoform, and potentially allows for the expression of an unusually large “N2BA-G” isoforms that result in very high titin compliance (Guo et al., 2012). In such studies, reducing the content of functioning RBM20 led to increased titin compliance and improved exercise tolerance (Methawasin et al., 2016; Slater et al., 2017). In humans, cardiac compliance also appears to be a strong predictor of exercise tolerance and predicts exercise tolerance better than heart failure status or ejection fraction (Meyer et al., 2004; Nagueh et al., 2004). However, with the exception of one study directly correlating the N2BA:N2B ratio with exercise tolerance in heart failure patients who later underwent a heart transplant (Nagueh et al., 2004), studies in humans are limited mechanistically due to constraints on gathering human tissues.

Clinical studies in humans continue to produce evidence that cardiac compliance is increased by exercise training and reduced by sedentary behaviors. The relationship between sedentary behaviors and cardiac compliance is observed in both patients with heart failure and otherwise healthy individuals (Edelmann et al., 2011; Howden et al., 2018). The molecular mechanisms underlying the increased compliance in these human studies have not been elucidated. Previous studies in small animal models have reported that the N2BA:N2B ratio does not differ between exercising and sedentary conditions. Thus, our data (Figure 1) is the first biochemical evidence that sedentary conditions reduce the titin N2BA:N2B ratio. The modest change in isoform ratios may be important, given that significant improvements in hazard ratios are observed only with modest amounts of activity and do not improve with increasing activity (Lavie et al., 2019).

The methodology may indicate why a difference in titin isoform content between sedentary and active rodents was not previously revealed. First, the exercise-related activity in the prior studies lasted just 4 weeks or less (Hidalgo et al., 2014; Muller et al., 2014; Slater et al., 2017). In contrast, the current study compared the hearts of rats that underwent 12 weeks of housing in sedentary conditions or active conditions with unrestricted access to a voluntary running wheel. Second, while both mice and rats show high content of the stiff N2B isoform, mice appear to have very low amounts of N2BA naturally, and the N2BA isoform is only observed in mice under extreme stress, such as in heart failure models (Wu et al., 2000). Third, the natural variances in the N2BA:N2B ratios in ours and previous studies suggest that the small sample sizes observed in previous studies (typically *n* = 6) were underpowered to observe any effect. Sampling analysis of our data suggests that more than 10 hearts per group were required to observe significant differences. Thus, the current study may be the first that provides both a sufficiently long intervention and sufficient sample size to report that sedentary conditions were associated with a decrease in the titin N2BA:N2B isoform ratio.

### Mechanisms to Modify Titin Isoform Content

The titin protein is encoded by a single gene, so investigating *titin* gene expression does not provide insight into mechanisms that alter isoform expression. However, the change in the N2BA:N2B ratio is likely driven by the splicing factor RBM20 (RNA Binding Motif 20 protein). Increasing content of RBM20 is associated with reductions in the proportions of the N2BA isoform (Guo et al., 2012, 2013; Methawasin et al., 2016; Zhu et al., 2017). Loss of RBM20 activity in rodent models results in increases in the content of the N2BA isoform or the induction of unusually large titin isoforms (Guo et al., 2012). RBM20 has been suggested as a therapeutic target to improve left ventricular stiffness and exercise tolerance (Methawasin et al., 2016). For example, a heterozygous, conditional disruption of RBM20 in mice increased titin-based compliance, which improved exercise tolerance (Methawasin et al., 2016). In contrast, homozygous mutant rats have reduced exercise tolerance compared to wild-type control rats (Guo et al., 2013), suggesting a J-curve in the relationship between exercise tolerance and RBM20 expression.

We investigated gene expression of both RBM20 and PTB4 (Guo et al., 2012; Dauksaite and Gotthardt, 2018) to evaluate if transcription of these proteins related to titin isoform content. We did not observe a significant decrease in *rbm20* mRNA expression in the hearts of exercising rats versus the sedentary controls (Figure 2A). *Rbm20* mRNA expression level did, however, correlate with the titin N2BA:N2B ratio (Figure 2B), suggesting that RBM20 may still contribute to the change in N2BA:N2B ratio, but that the expression level changes are modest. RBM20 activity might also be influenced by PTB4, which was recently proposed as an inhibitor of RBM20’s splicing of *titin* mRNA (Dauksaite and Gotthardt, 2018). We found no change in *ptb* gene expression. While our data suggest that chronic exercise does not alter *ptb* expression, it is possible that our inability to observe changes is limited by the primer used, since it cannot differentiate between the PTB isoforms (Wollerton et al., 2001).

Other regulators of RBM20 might also be important to analyze in future studies. For example, RBM20 is downstream of the insulin signaling pathway (Zhu et al., 2017). Exercise itself can modify insulin sensitivity and glucose uptake (Kirwan et al., 2009) so changes in RBM20 may be dependent on subtle changes on this PI3K/AKT dependent pathway. Similarly, exercise might modify the phosphorylation of RBM20 or alter its splicing activity (Zhu et al., 2017; Murayama et al., 2018). Thus, while we have reported a correlation between *rbm20* gene expression and the titin N2BA:N2B ratio, the precise activity-dependent mechanism(s) that might modify titin isoform expression is not fully elucidated. Thus, future studies into protein content and activity of splicing factors such as RBM20 and PTB4 may be warranted.

### Post-translational Modifications

Titin compliance can be directly modified by a wide array of post-translational modifications (Koser et al., 2019), of which phosphorylation is the most well characterized. For example, a single bout of treadmill exercise reduced compliance by increasing PKC and reducing PKA phosphorylation of titin (Muller et al., 2014). A 3- or 4-week exposure to voluntary running wheel exercise results in the reverse, i.e., reductions in PKC phosphorylation and increases in PKA phosphorylation, which is consistent with an increase in compliance (Hidalgo et al., 2014; Slater et al., 2017). PKG, ERK, and CaMKII are other known modifiers of cardiac stiffness (Koser et al., 2019). When PKG, ERK, and CaMKII bind to the N2B element of titin, compliance increases, and CaMKII can play a dual role by also phosphorylating the PEVK element to increase passive tension like PKC. In mice, PKG was not shown to be different after 4-weeks of exercise (Hidalgo et al., 2014), but the effect of long term exercise on ERK and CamKII phosphorylation targets on titin is not yet described. Similarly, the effect of exercise on other modifiers of titin such as oxidative stress (including disulphide bonding and glutathionylation) (Alegre-Cebollada et al., 2014; Koser et al., 2019) are not known.

In the current study, we assessed total phosphorylation using ProQ Diamond staining, which has previously helped identify titin phosphorylation sites, but we observed no difference between the sedentary and active group. Two challenges of ProQ Diamond staining are first that it may not reflect PKC phosphorylation of the PEVK element of titin (Hudson et al., 2011) and second that more than 300 total putative phosphorylation sites on titin have been detected by mass spectrometry (Koser et al., 2019) meaning it is not specific. Nonetheless, we might speculate that, consistent with results reported in 4-week studies, exercise is likely to be associated with a reduction in PKC phosphorylation of the PEVK element, along with trends in increases of PKA of the N2B element (Hidalgo et al., 2014; Slater et al., 2017). Further, exercise has been associated with increasing activity of ERK and CaMKII (Hamdani et al., 2013; Perkin et al., 2015; Koser et al., 2019), supporting a role for post-translational modifications to increase compliance. While not feasible with the current samples, direct measurement of myocardial compliance and its response to kinase specific (de)phosphorylation would be a reasonable future study.

The current study focused on chronic changes to titin isoform switching. It is not known if non-titin related phosphorylation impacts chronic changes in stiffness such as with the phosphorylation of RBM20, as noted above (Murayama et al., 2018). The resultant change in titin isoform content by RBM20 regulation may itself modulate the impact of phosphorylation on titin compliance. Published works suggest that the two isoforms may be differentially phosphorylated and that phosphorylation by PKA has a reduced effect in tissues with more N2BA (Fukuda et al., 2005; Borbely et al., 2009; Koser et al., 2019). Thus, even a modest increase in the content of the more compliant N2BA isoform may cause a multiplicative increase in compliance because of changes in the phosphorylation status.

### Perspective and Summary

Sedentary behavior is associated with significant general and cardiovascular health risks and reduced exercise tolerance (Blair et al., 1995; Bassett et al., 2010; Martin et al., 2010; Lalande et al., 2017; Howden et al., 2018; Lavie et al., 2019). In laboratory animals, voluntary wheel running is more equivalent to the natural active state whereas standard caging is more similar to sedentary behaviors (Meijer and Robbers, 2014; Booth et al., 2017). We evaluated whether titin isoform content might be modified by sedentary conditions in rats. Our study is the first to report that sedentary conditions reduce the content of long, compliant titin isoforms in rat left ventricles. This result suggests that reduced cardiac compliance, which is associated with sedentary behavior, may be alleviated by chronic exercise by altering the titin isoform content.

## Nomenclature

SED Samples from rats after 12 weeks of sedentary housing
EX Samples from rats after 12 weeks of voluntary exercise housing
N2B The smaller, less compliant cardiac isoform of the giant titin protein
N2BA The larger, more compliant isoform of the giant titin protein
N2BA:N2B Ratio of titin isoform content
MHC Myosin Heavy Chain
RBM20 RNA binding motif 20; splicing factor for *titin* mRNA
PTB Polypyrimidine Tract Binding protein; a putative splicing factor for *titin* mRNA

## Data Availability Statement

The datasets generated for this study are available on request to the corresponding author.

## Ethics Statement

The animal study was reviewed and approved by the Institutional Animal Care and Use Committee Wayne State University.

## Author Contributions

All authors contributed to data acquisition, data analysis, manuscript writing and editing, and approved the submitted version of the manuscript.

## Conflict of Interest

The authors declare that the research was conducted in the absence of any commercial or financial relationships that could be construed as a potential conflict of interest.

## References

[B1] Alegre-Cebollada J. Kosuri P. Giganti D. Eckels E. Rivas-Pardo J. A. Hamdani N. (2014). S-glutathionylation of cryptic cysteines enhances titin elasticity by blocking protein folding. *Cell* 156 1235–1246. 10.1016/j.cell.2014.01.056 24630725 PMC3989842

[B2] Bassett D. R. Jr. Freedson P. Kozey S. (2010). Medical hazards of prolonged sitting. *Exerc. Sport Sci. Rev.* 38 101–102. 10.1097/jes.0b013e3181e373ee 20577056

[B3] Blair S. N. Kohl H.W. 3rd Barlow C. E. Paffenbarger R. S. Jr. Gibbons L. W. Macera C. A. (1995). Changes in physical fitness and all-cause mortality. a prospective study of healthy and unhealthy men. *JAMA* 273 1093–1098. 10.1001/jama.273.14.1093 7707596

[B4] Booth F. W. Roberts C. K. Thyfault J. P. Ruegsegger G. N. Toedebusch R. G. (2017). Role of inactivity in chronic diseases: evolutionary insight and pathophysiological mechanisms. *Physiol. Rev.* 97 1351–1402. 10.1152/physrev.00019.2016 28814614 PMC6347102

[B5] Borbely A. Falcao-Pires I. Van Heerebeek L. Hamdani N. Edes I. Gavina C. (2009). Hypophosphorylation of the stiff N2B titin isoform raises cardiomyocyte resting tension in failing human myocardium. *Circ. Res.* 104 780–786. 10.1161/CIRCRESAHA.108.193326 19179657

[B6] Buck D. Hudson B. D. Ottenheijm C. A. Labeit S. Granzier H. (2010). Differential splicing of the large sarcomeric protein nebulin during skeletal muscle development. *J. Struct. Biol.* 170 325–333. 10.1016/j.jsb.2010.02.014 20176113 PMC2856706

[B7] Chung C. S. Granzier H. L. (2011). Contribution of titin and extracellular matrix to passive pressure and measurement of sarcomere length in the mouse left ventricle. *J. Mol. Cell. Cardiol.* 50 731–739. 10.1016/j.yjmcc.2011.01.005 21255582 PMC3057392

[B8] Chung C. S. Hutchinson K. R. Methawasin M. Saripalli C. Smith J. E. III Hidalgo C. G. (2013). Shortening of the elastic tandem immunoglobulin segment of titin leads to diastolic dysfunction. *Circulation* 128 19–28. 10.1161/CIRCULATIONAHA.112.001268 23709671 PMC3822017

[B9] Dauksaite V. Gotthardt M. (2018). Molecular basis of titin exon exclusion by RBM20 and the novel titin splice regulator PTB4. *Nucleic Acids Res.* 46 5227–5238. 10.1093/nar/gky165 29518215 PMC6007684

[B10] Edelmann F. Gelbrich G. Dungen H. D. Frohling S. Wachter R. Stahrenberg R. (2011). Exercise training improves exercise capacity and diastolic function in patients with heart failure with preserved ejection fraction: results of the Ex-DHF (Exercise training in Diastolic Heart Failure) pilot study. *J. Am. Coll. Cardiol.* 58 1780–1791. 10.1016/j.jacc.2011.06.054 21996391

[B11] Freiburg A. Trombitas K. Hell W. Cazorla O. Fougerousse F. Centner T. (2000). Series of exon-skipping events in the elastic spring region of titin as the structural basis for myofibrillar elastic diversity. *Circ. Res.* 86 1114–1121. 10.1161/01.res.86.11.1114 10850961

[B12] Fujimoto N. Prasad A. Hastings J. L. Bhella P. S. Shibata S. Palmer D. (2012). Cardiovascular effects of 1 year of progressive endurance exercise training in patients with heart failure with preserved ejection fraction. *Am. Heart J.* 164 869–877. 10.1016/j.ahj.2012.06.028 23194487 PMC3727249

[B13] Fukuda N. Wu Y. Nair P. Granzier H. L. (2005). Phosphorylation of titin modulates passive stiffness of cardiac muscle in a titin isoform-dependent manner. *J. Gen. Physiol.* 125 257–271. 10.1085/jgp.200409177 15738048 PMC2234012

[B14] Granzier H. L. Irving T. C. (1995). Passive tension in cardiac muscle: contribution of collagen, titin, microtubules, and intermediate filaments. *Biophys. J.* 68 1027–1044. 10.1016/s0006-3495(95)80278-x 7756523 PMC1281826

[B15] Granzier H. L. Labeit S. (2004). The giant protein titin: a major player in myocardial mechanics, signaling, and disease. *Circ. Res.* 94 284–295. 10.1161/01.res.0000117769.88862.f8 14976139

[B16] Guo W. Pleitner J. M. Saupe K. W. Greaser M. L. (2013). Pathophysiological defects and transcriptional profiling in the RBM20-/- rat model. *PLoS One* 8:e84281. 10.1371/journal.pone.0084281 24367651 PMC3868568

[B17] Guo W. Schafer S. Greaser M. L. Radke M. H. Liss M. Govindarajan T. (2012). RBM20, a gene for hereditary cardiomyopathy, regulates titin splicing. *Nat. Med.* 18 766–773. 10.1038/nm.2693 22466703 PMC3569865

[B18] Hamdani N. Krysiak J. Kreusser M. M. Neef S. Dos Remedios C. G. Maier L. S. (2013). Crucial role for Ca2(+)/calmodulin-dependent protein kinase-II in regulating diastolic stress of normal and failing hearts via titin phosphorylation. *Circ. Res.* 112 664–674. 10.1161/CIRCRESAHA.111.300105 23283722

[B19] Hidalgo C. Saripalli C. Granzier H. L. (2014). Effect of exercise training on post-translational and post-transcriptional regulation of titin stiffness in striated muscle of wild type and IG KO mice. *Arch. Biochem. Biophys.* 552-553 100–107. 10.1016/j.abb.2014.02.010 24603287

[B20] Howden E. J. Sarma S. Lawley J. S. Opondo M. Cornwell W. Stoller D. (2018). Reversing the cardiac effects of sedentary aging in middle age-a randomized controlled trial: implications for heart failure prevention. *Circulation* 137 1549–1560. 10.1161/CIRCULATIONAHA.117.030617 29311053 PMC5893372

[B21] Hudson B. Hidalgo C. Saripalli C. Granzier H. (2011). Hyperphosphorylation of mouse cardiac titin contributes to transverse aortic constriction-induced diastolic dysfunction. *Circ. Res.* 109 858–866. 10.1161/CIRCRESAHA.111.246819 21835910 PMC3191198

[B22] Kirwan J. P. Solomon T. P. Wojta D. M. Staten M. A. Holloszy J. O. (2009). Effects of 7 days of exercise training on insulin sensitivity and responsiveness in type 2 diabetes mellitus. *Am. J. Physiol. Endocrinol. Metab.* 297 E151–E156. 10.1152/ajpendo.00210.2009 19383872 PMC2711659

[B23] Koser F. Loescher C. Linke W. A. (2019). Posttranslational modifications of titin from cardiac muscle: how, where, and what for? *FEBS J.* 286 2240–2260. 10.1111/febs.14854 30989819 PMC6850032

[B24] Lahmers S. Wu Y. Call D. R. Labeit S. Granzier H. (2004). Developmental control of titin isoform expression and passive stiffness in fetal and neonatal myocardium. *Circ. Res.* 94 505–513. 10.1161/01.res.0000115522.52554.86 14707027

[B25] Lalande S. Mueller P. J. Chung C. S. (2017). The link between exercise and titin passive stiffness. *Exp. Physiol.* 102 1055–1066. 10.1113/EP086275 28762234 PMC5578882

[B26] Lavie C. J. Ozemek C. Carbone S. Katzmarzyk P. T. Blair S. N. (2019). Sedentary behavior, exercise, and cardiovascular health. *Circ. Res.* 124 799–815. 10.1161/CIRCRESAHA.118.312669 30817262

[B27] Li S. Guo W. Schmitt B. M. Greaser M. L. (2012). Comprehensive analysis of titin protein isoform and alternative splicing in normal and mutant rats. *J. Cell. Biochem.* 113 1265–1273. 10.1002/jcb.23459 22105831 PMC6696936

[B28] Livak K. J. Schmittgen T. D. (2001). Analysis of relative gene expression data using real-time quantitative PCR and the 2(-Delta Delta C(T)) Method. *Methods* 25 402–408. 10.1006/meth.2001.1262 11846609

[B29] Martin B. Ji S. Maudsley S. Mattson M. P. (2010). “Control” laboratory rodents are metabolically morbid: why it matters. *Proc. Natl. Acad. Sci. U.S.A.* 107 6127–6133. 10.1073/pnas.0912955107 20194732 PMC2852022

[B30] Meijer J. H. Robbers Y. (2014). Wheel running in the wild. *Proc. Biol. Sci.* 281:20140210. 10.1098/rspb.2014.0210 24850923 PMC4046404

[B31] Methawasin M. Strom J. G. Slater R. E. Fernandez V. Saripalli C. Granzier H. (2016). Experimentally increasing the compliance of titin through RNA binding Motif-20 (RBM20) inhibition improves diastolic function in a mouse model of heart failure with preserved ejection fraction. *Circulation* 134 1085–1099. 10.1161/circulationaha.116.023003 27630136 PMC5069184

[B32] Meyer T. E. Karamanoglu M. Ehsani A. A. Kovacs S. J. (2004). Left ventricular chamber stiffness at rest as a determinant of exercise capacity in heart failure subjects with decreased ejection fraction. *J. Appl. Physiol.* 97 1667–1672. 10.1152/japplphysiol.00078.2004 15208299

[B33] Mischel N. A. Mueller P. J. (2011). (In)activity-dependent alterations in resting and reflex control of splanchnic sympathetic nerve activity. *J. Appl. Physiol.* 111 1854–1862. 10.1152/japplphysiol.00961.2011 21979802 PMC3233897

[B34] Muller A. E. Kreiner M. Kotter S. Lassak P. Bloch W. Suhr F. (2014). Acute exercise modifies titin phosphorylation and increases cardiac myofilament stiffness. *Front. Physiol.* 5:449. 10.3389/fphys.2014.00449 25477822 PMC4238368

[B35] Murayama R. Kimura-Asami M. Togo-Ohno M. Yamasaki-Kato Y. Naruse T. K. Yamamoto T. (2018). Phosphorylation of the RSRSP stretch is critical for splicing regulation by RNA-binding motif protein 20 (RBM20) through nuclear localization. *Sci. Rep.* 8:8970. 10.1038/s41598-018-26624-w 29895960 PMC5997748

[B36] Nagueh S. F. Shah G. Wu Y. Torre-Amione G. King N. M. Lahmers S. (2004). Altered titin expression, myocardial stiffness, and left ventricular function in patients with dilated cardiomyopathy. *Circulation* 110 155–162. 10.1161/01.cir.0000135591.37759.af 15238456

[B37] Perkin J. Slater R. Del Favero G. Lanzicher T. Hidalgo C. Anderson B. (2015). Phosphorylating titin’s cardiac N2B element by ERK2 or CaMKIIdelta lowers the single molecule and cardiac muscle force. *Biophys. J.* 109 2592–2601. 10.1016/j.bpj.2015.11.002 26682816 PMC4701010

[B38] Rio D. C. Ares M. Jr. Hannon G. J. Nilsen T. W. (2010). Purification of RNA using TRIzol (TRI reagent). *Cold Spring Harb. Protoc.* 2010:dbrot5439. 10.1101/pdb.prot5439 20516177

[B39] Slater R. E. Strom J. G. Granzier H. (2017). Effect of exercise on passive myocardial stiffness in mice with diastolic dysfunction. *J. Mol. Cell. Cardiol.* 108 24–33. 10.1016/j.yjmcc.2017.04.006 28476659

[B40] Sternberg S. R. (1983). Biomedical image processing. *Computer* 16 22–34.

[B41] Trombitas K. Wu Y. Labeit D. Labeit S. Granzier H. (2001). Cardiac titin isoforms are coexpressed in the half-sarcomere and extend independently. *Am. J. Physiol. Heart Circ. Physiol.* 281 H1793–H1799. 11557573 10.1152/ajpheart.2001.281.4.H1793

[B42] Wollerton M. C. Gooding C. Robinson F. Brown E. C. Jackson R. J. Smith C. W. (2001). Differential alternative splicing activity of isoforms of polypyrimidine tract binding protein (PTB). *RNA* 7 819–832. 10.1017/s1355838201010214 11421360 PMC1370133

[B43] Wu Y. Cazorla O. Labeit D. Labeit S. Granzier H. (2000). Changes in titin and collagen underlie diastolic stiffness diversity of cardiac muscle. *J. Mol. Cell. Cardiol.* 32 2151–2162. 11112991 10.1006/jmcc.2000.1281

[B44] Zhu C. Yin Z. Tan B. Guo W. (2017). Insulin regulates titin pre-mRNA splicing through the PI3K-Akt-mTOR kinase axis in a RBM20-dependent manner. *Biochim. Biophys. Acta Mol. Basis Dis.* 1863 2363–2371. 10.1016/j.bbadis.2017.06.023 28676430 PMC5547897

[B45] Zhu C. Q. Guo W. (2017). Detection and quantification of the giant protein titin by SDS-agarose gel electrophoresis. *Methodsx* 4 320–327. 10.1016/j.mex.2017.09.007 29872636 PMC5986978

